# Diagnosis of Crohn’s disease and ulcerative colitis using the microbiome

**DOI:** 10.1186/s12866-023-03084-5

**Published:** 2023-11-11

**Authors:** Da-Yeon Kang, Jong-Lyul Park, Min-Kyung Yeo, Sang-Bum Kang, Jin-Man Kim, Ju Seok Kim, Seon-Young Kim

**Affiliations:** 1 Department of New Drug Development, Graduate School of New Drug Discovery and Development, Chungnam National University, Daejeon, Korea; 2 Disease Target Structure Research Center, Korea Research Institute of Bioscience and Biotechnology (KRIBB), Daejeon, Korea; 3 Aging Convergence Research Center, Korea Research Institute of Bioscience and Biotechnology (KRIBB), Daejeon, Korea; 4 Department of Pathology, Chungnam National University School of Medicine, Munwha-Ro 266, Daejeon, 35015 Korea; 5 Department of Internal Medicine, Division of Gastroenterology, Daejeon St. Mary’s Hospital, College of Medicine, The Catholic University of Korea, Daejeon, Korea; 6 Departments of Internal Medicine, Chungnam National University School of Medicine, Daejeon, Korea; 7 Korea Bioinformation Center, Korea Research Institute of Bioscience and Biotechnology (KRIBB), Daejeon, Korea

**Keywords:** Gut microbiome, Inflammatory bowel disease, Crohn’s disease, Ulcerative colitis, Machine learning, Whole metagenome shotgun (WMS) sequencing

## Abstract

**Background:**

Inflammatory bowel disease (IBD) is a multifactorial chronic inflammatory disease resulting from dysregulation of the mucosal immune response and gut microbiota. Crohn's disease (CD) and ulcerative colitis (UC) are difficult to distinguish, and differential diagnosis is essential for establishing a long-term treatment plan for patients. Furthermore, the abundance of mucosal bacteria is associated with the severity of the disease. This study aimed to differentiate and diagnose these two diseases using the microbiome and identify specific biomarkers associated with disease activity.

**Results:**

Differences in the abundance and composition of the microbiome between IBD patients and healthy controls (HC) were observed. Compared to HC, the diversity of the gut microbiome in patients with IBD decreased; the diversity of the gut microbiome in patients with CD was significantly lower. Sixty-eight microbiota members (28 for CD and 40 for UC) associated with these diseases were identified. Additionally, as the disease progressed through different stages, the diversity of the bacteria decreased. The abundances of *Alistipes shahii* and *Pseudodesulfovibrio aespoeensis* were negatively correlated with the severity of CD, whereas the abundance of *Polynucleobacter wianus* was positively correlated. The severity of UC was negatively correlated with the abundance of *A. shahii*, *Porphyromonas asaccharolytica and Akkermansia muciniphilla,* while it was positively correlated with the abundance of *Pantoea candidatus pantoea carbekii*. A regularized logistic regression model was used for the differential diagnosis of the two diseases. The area under the curve (AUC) was used to examine the performance of the model. The model discriminated UC and CD at an AUC of 0.873 (train set), 0.778 (test set), and 0.633 (validation set) and an area under the precision-recall curve (PRAUC) of 0.888 (train set), 0.806 (test set), and 0.474 (validation set).

**Conclusions:**

Based on fecal whole-metagenome shotgun (WMS) sequencing, CD and UC were diagnosed using a machine-learning predictive model. Microbiome biomarkers associated with disease activity (UC and CD) are also proposed.

**Supplementary Information:**

The online version contains supplementary material available at 10.1186/s12866-023-03084-5.

## Introduction

Inflammatory bowel disease (IBD) is a multifactorial disease that results in chronic intestinal inflammation due to the dysregulation of immune responses. More than 3.5 million patients in the United States and Europe have IBD, which is becoming more common worldwide [1].

The human gut microbiome plays an important role in nutrient metabolism, pathogen protection, and immune system development. It is generally accepted that the development and progression of IBD are more closely associated with the gut microbiome [2–6]. In IBD, genetic and environmental factors such as an altered gut microbiome and enhanced intestinal permeability play roles in deregulating intestinal immunity, eventually leading to gastrointestinal damage [7, 8]. IBD includes Crohn's disease (CD) and ulcerative colitis (UC). Although the distribution, location, and histology of the inflammatory site vary between these two diseases, approximately 10–15% of patients exhibit the same pattern [9, 10]. Moreover, it is difficult to classify the state of the disease because the clinical and pathological characteristics overlap [9]. A clear differentiation between these two disorders must be made to develop an effective treatment plan.

As CD and UC are progressive conditions, determining their severity is essential for selecting the best course of treatment [11, 12]. According to Swidsinski et al., the concentration of mucosal-associated bacteria was related to the severity of the disease [13, 14]. Potential variations in the microbial composition related to disease activity may serve as markers for disease monitoring. This study investigated biomarkers for differential diagnosis and identified distinctive microbiomes associated with disease activity.

## Results

### Clinical statistics of the study population

In this study, 482 samples were obtained from 50 healthy controls (HC), 173 patients with CD, and 259 patients with UC. The demographic and clinical characteristics of the participants are presented in Table 1. There were differences in sex, age, and body mass index (BMI) between CD and UC patients, but no differences in family history. Table 2 shows the basic characteristics of patients with CD according to disease activity groups. Of the 173 patients, we excluded 82 who received biologics, 4 who underwent surgery, and 18 who underwent both biologics and surgery. Of the 69 patients with CD, 49 had mild CD, 19 moderate CD, and one severe CD. There were no differences in sex, age, BMI, family history, or smoking status between the mild and moderate patient groups. Table 3 shows the basic characteristics of the patients with UC according to their disease activity. Of the 259 patients, 61 who received biologics were excluded. Of 198 patients with UC, 162 had mild UC, 25 had moderate UC, and 11 had severe UC. Sex, age, BMI, and smoking status were not significantly different in the mild, moderate, and severe groups of patients, but there was a significant difference in family history (Table 3).

**Table 1:** Clinicopathological characteristics used in this study

Group	HC	CD	UC	*p*-value
Number	50	173	259	
Gender				< 0.001
Male	17 (34.0%)	133 (76.9%)	155 (59.8%)	
Female	33 (66.0%)	40 (23.1%)	104 (40.2%)	
Age, median years (range)	38 (19—70)	30 (14—83)	49 (19—84)	< 0.001
BMI, median BMI (range)	23.7 (16.2—32.8)	20.4 (12.4—36.5)	22.68 (14.06—38.2)	< 0.001
Family history	-			0.493
Yes		6 (3.5%)	15 (5.8%)	
No		149 (86.1%)	221(85.3%)	
Un-known		18 (10.4%)	23 (8.9%)	
Smoking history				< 0.001
Never	36 (72.0%)	102 (59.0%)	125 (48.3%)	
Past smoker	11 (22.0%)	26 (15.0%)	96 (37.1%)	
Current smoker	3 (6.0%)	26 (15.0%)	18 (6.9%)	
Un-known	0 (0.0%)	19 (11.0%)	20 (7.7%)

**Table 2:** **A** basic characteristic of CD patients according to disease activity groups

Group	Mild	Moderate	*p*-value
Number	49	19	
Gender			0.153
Male	36 (73.5%)	17 (89.5%)	
Female	13 (26.5%)	2 (10.5%)	
Age, median years (range)	30 (19–83)	29 (14–56)	0.341
BMI, median BMI (range)	22.2 (14.5–36.5)	20.1 (13.1–31.5)	0.110
Family history			0.446
Yes	2 (4.1%)	0 (0.0%)	
No	41 (83.7%)	18 (94.7%)	
Un-known	6 (12.2%)	1 (5.3%)	
Smoking history			0.774
Never	27 (55.1%)	13 (68.4%)	
Past smoker	7 (14.3%)	2 (10.5%)	
Current smoker	9 (18.4%)	2 (10.5%)	
Un-known	6 (12.2%)	2 (10.5%)

**Table 3:** A basic characteristic of UC patients according to disease activity groups

Group	Mild	Moderate	Severe	*p*-value
Number	162	25	11	
Gender				0.520
Male	102 (63.0%)	13 (52.0%)	6 (54.5%)	
Female	60 (37.0%)	12 (48.0%)	5 (45.5%)	
Age, median years (range)	49 (19–84)	43 (21–71)	47 (24–67)	0.396
BMI, median BMI (range)	22.8 (14.5–38.2)	23.9 (16.9–30)	19.7 (17.6–26.4)	0.176
Family history				0.020
Yes	8 (4.9%)	0 (0.0%)	3 (27.3%)	
No	138 (85.2%)	22 (88.0%)	7 (63.6%)	
Un-known	16 (9.9%)	3 (12.0%)	1 (9.1%)	
Smoking history				0.806
Never	77 (47.5%)	14 (56.0%)	4 (36.4%)	
Past smoker	59 (36.4%)	7 (28.0%)	4 (36.4%)	
Current smoker	13 (8.0%)	1 (4.0%)	1 (9.1%)	
Un-known	13 (8.0%)	3 (12.0%)	2 (18.2%)	

### Association of the enterotypes with IBD

Enterotypes are used to categorize people according to their gut microbiomes and as potential biomarkers of the healthy human intestine. Enterotypes have recently attracted attention in predicting their relationships with diseases [15]. So, we examined the relationships between enterotypes and the health status of our cohort. Principal Coordinate Analysis (PCoA) of the gut microbiome of 50 HC and 432 IBD patients revealed three groups (Fig. S1A). The abundance of *Bifidobacterium* was similar between enterotype 2 and 3, although that of *Bacteroides* was higher in enterotype 2 than in enterotype 1, and that of *Faecalibacterium* was similar between enterotype 1 and 3 (Fig. S1B). 42.5% of the samples were grouped into the enterotype 2 cluster (*Faecalibacterium*), followed by 32.1% of the enterotype 1 cluster (*Bifidobacterium*) and 25.3% of the enterotype 3 cluster (*Bacteroides*). The analysis showed that their distribution in HC and IBD was significantly different, as shown in Table S1 (Fisher’s exact test, *p* < 0.05). The enterotype with a *Faecalibacterium* predominance was underrepresented in the HC (74% vs 37% vs 40%).

### Differences in the taxonomic composition of the gut microbiome in patients with CD and UC

**Fig. 1:**
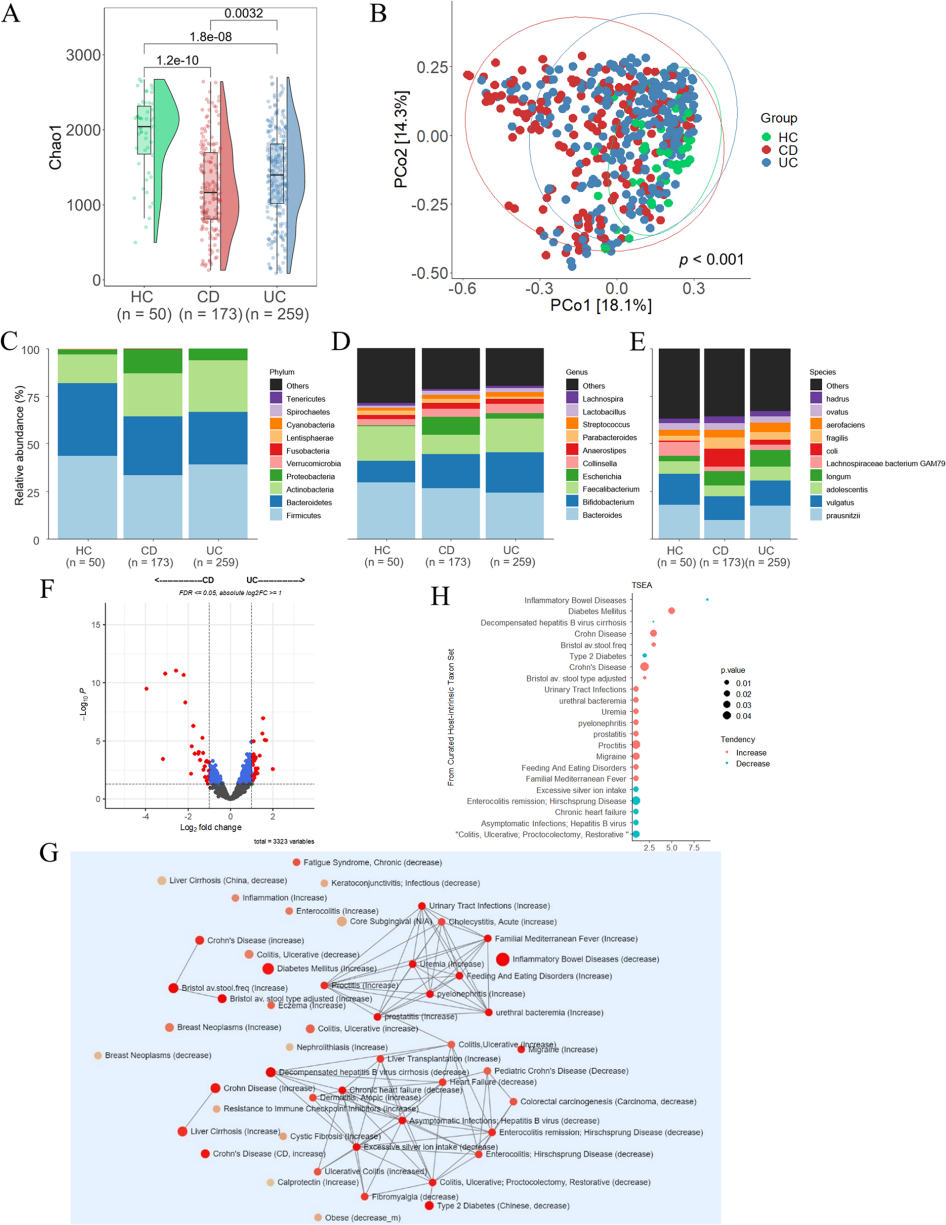
Diversity and distribution of microbiomes in healthy control (HC) and IBD (CD and UC) patient groups. **A** Alpha diversity was calculated as the Chao1 index; (**B**) Beta diversity was calculated as Bray–Curtis dissimilarity. PCoA was used to display the microbiome space among the groups. **C**, **D**, **E** Comparison of relative taxa abundance among HC, CD, and UC at the phylum (**C**), genus (**D**), and species (**E**) levels. The taxonomic biomarkers were identified using LinDA (FDR <  = 0.05, |log2FC|> = 1). **F** Volcano plot of the composition of the intestinal microbial community at the species level. **G** Taxon Set Enrichment Analysis (TSEA) results showing the network modules of enriched terms. **H** Taxon set terms representing the association of human diseases with the known composition of the groups (*p* <  = 0.05)

The abundance tables were rarefied to 1,222,561 sequences per sample by random subsampling using the phyloseq R package [16]. To evaluate the alpha diversity, we calculated the Chao1 diversity index, which considers the number, uniformity, and abundance of taxa observed in each sample. The alpha diversity indices were lower in patients with IBD than healthy controls. The Chao1 diversity index was significantly lower in patients with UC and CD than in HC and was significantly lower in patients with CD than in UC patients (Fig. 1A). Beta diversity was measured as the Bray–Curtis distance, which was visualized using principal coordinate analysis (PCoA) (Fig. 1B). The relative abundance of the gut microbiome was compared among the groups (HC, CD, and UC). The following percentages in parentheses are in the order of HC > CD > UC. Compared to HC, IBD patients had significantly higher abundances of phylum *Actinobacteria* (15.22, 22.77, and 27.13%). It is also more abundant in patients with UC than those with CD. In contrast, *Firmicutes* (43.60, 33.75, and 39.14%) decreased in patients with IBD compared to HC and were more reduced in patients with CD than in UC patients (Fig. 1C and Fig. S2A). At the genus level, *Escherichia* (0.64, 9.48, and 2.67%) increased in patients with IBD compared to HC and was significantly higher in patients with CD than in UC patients. In contrast, *Faecalibacterium* (18.13, 10.04, and 17.54%) was reduced in patients with IBD compared to that in HC; it decreased significantly in patients with CD compared to that in UC patients (Fig. 1D and Fig. S2B). At the species level, *Lachnospiraceae bacterium GAM79* (7.53, 2.37, and 2.97%) was lower in patients with IBD than in HC, and patients with CD than in UC patients. *E. coli* (0.67, 9.45, and 2.68%) was more abundant in patients with IBD and significantly more abundant in CD patients (Fig. 1E and Fig. S2C). Linear models for differential abundance analysis (LinDA) was used to compare the predominance of communities between groups, and significant differences in microbial abundance were observed between the CD and UC groups. Sixty-eight microbiotas were identified at the species level, among which 28 were over-represented in the CD group and 40 were over-represented in the UC group (Fig. 1F, FDR <  = 0.05, absolute log2FC >  = 1). *Shigella dysenteriae, Erysipelotrichaceae bacterium|46, E. coli, and Escherichia marmotae* were more abundant in the CD group, whereas *Bacillus vallismortis, Lactobacillus ruminis, Alicycliphilus dentirficans,* and *Lachnospira eligens* were more abundant in the UC group (Table S2). Taxon set enrichment analysis (TSEA) was performed to identify the taxon sets associated with host intrinsic factors. We identified 23 disease-related terms that were significant in the curated set of host intrinsic factors, including decompensated hepatitis B virus cirrhosis, IBD, type 2 diabetes, chronic heart failure, excessive silver ion intake, and familial Mediterranean fever (Fig. 1G and H).

### Metabolic functions of the different gut microbiome

The HUMAnN3 tool was used to explore the potential functions of the microbial community. Subsequently, we compared the differentially enriched MetaCyc pathways and KEGG Orthology (KO) terms using LinDA. A significant difference in the MetaCyc pathway and KO terms was observed between patients with CD and UC. Sixty-eight differentially abundant MetaCyc pathways were identified. Among these, 66 pathways (phytate degradation I, mannosylglycerate biosynthesis I, and 2-methylcitrate cycle I etc.) were enriched in CD patients. In comparison, only two pathways (super pathways of polyamine biosynthesis II and superpathway ofmenaquinol-8 biosynthesis II) were enriched in UC patients (FDR <  = 0.05 and |log2FC|> = 1) (Table S3). In the KOs, 1,242 differentially enriched KO terms were found, and 1153 KO terms (FKBP-type peptidyl-prolyl cis–trans isomerase SlpA, biopolymer transport protein ExbD, and trehalose 6-phosphate phosphatase [EC:3.1.3.12]) were enriched in CD patients. In contrast, 89 KO terms (hypothetical protein, stage V sporulation protein AB, and multidrug/hemolysin transport system permease protein) were enriched in UC patients (FDR <  = 0.05 and |log2FC|> = 1) (Table S4).

### Alteration in gut microbiome according to the activity of CD

**Fig. 2:**
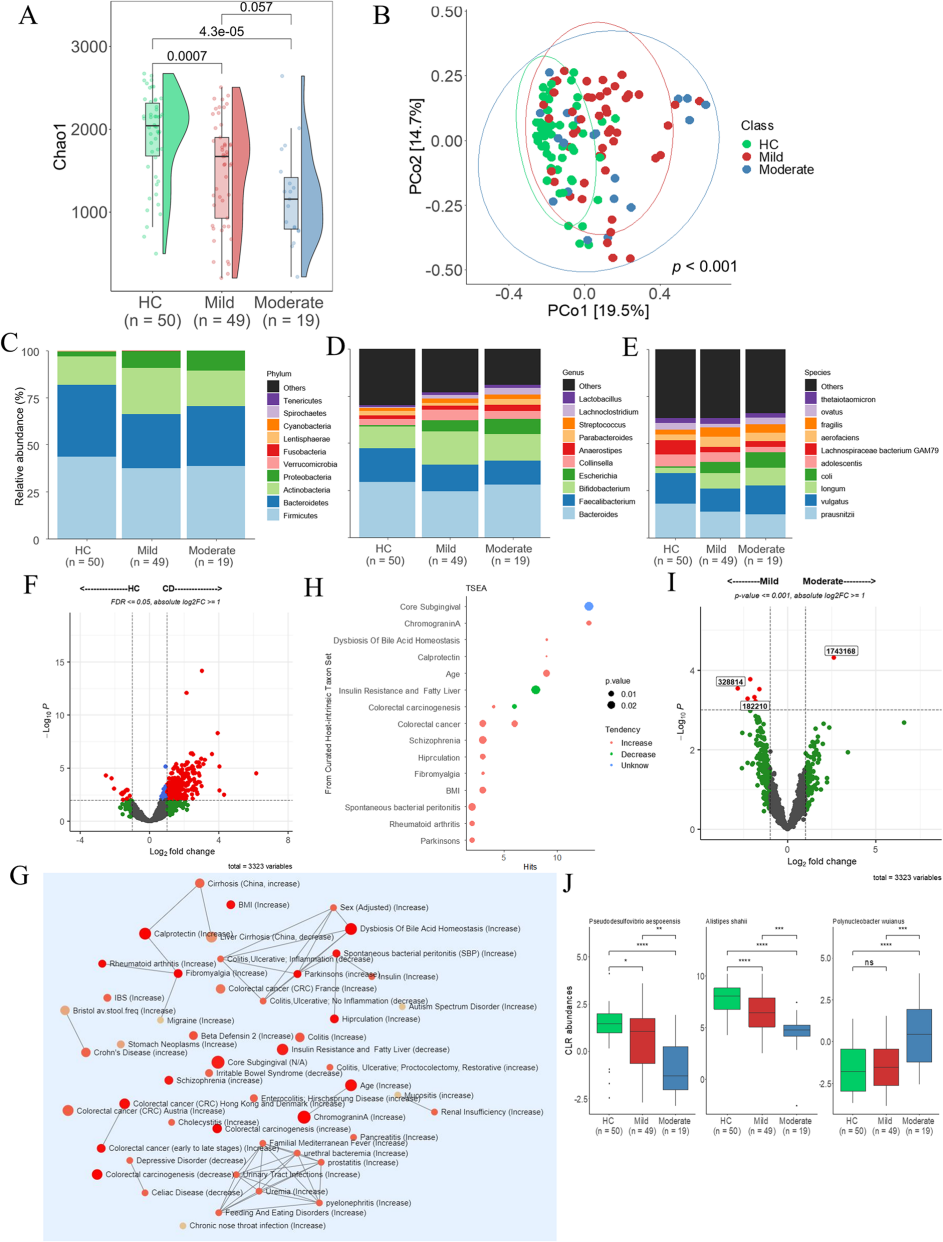
Diversity and distribution of microbiomes in CD subgroups. **A** Measurement of the Chao1 index of community richness. **B** PCoA showing differences in microbial composition by the Bray–Curtis index. **C**, **D**, **E** Taxonomic composition of the gut microbiome in the levels of CD-active group at the phylum (**C**), genus (**D**), and species (**E**). **F** Difference in microbial abundances between HC and CD (FDR <  = 0.05, |log2FC|> = 1). **G** TSEA network modules displayed by TSEA. **H** The TSEA results show 17 host-intrinsic terms (*p* <  = 0.05). **I** Comparison of the microbial composition between mild and moderate (*p* <  = 0.001, absolute log2FC >  = 1) at the species level. **J** Signatures of the gut microbiota related to CD activity. Box plots represent the median and interquartile range. The significances are represented as follows: ****p <  = 0.0001; ***p <  = 0.001; **p <  = 0.01; *p <  = 0.05; and ns, *p* > 0.05

The analysis was performed using the same pipeline as in the previous analysis. The alpha diversity index of the mild and moderate CD groups decreased more than that of the HC group. However, the difference between the mild and moderate groups was not significant. By analyzing beta diversity using Bray–Curtis and PCoA, we found significant differences in the microbial communities among the HC, mild, and moderate groups (Fig. 2A and B). The microbial composition of relative abundance differed between HC and CD but was similar in the mild and moderate groups. The following percentages in parentheses are in the order of HC > mild > moderate groups. *Verruciomicrobia* (0.27, 0.05, and 0.01%) and Cyanobacteria (0.04, 0.03, and 0.02%) at the phylum level were lower in the mild and moderate CD groups than in the HC group (Fig. 2C and Fig. S3A). At the genus level, *Escherichia* coli (0.64, 5.97, and 8.03%) increased in the mild and moderate groups compared to the HC group, but the difference between the mild and moderate groups was not significant (Fig. 2D and Fig. S3B). At the species level, *E. coli* (0.67, 5.99, and 8.07%) increased in the mild and moderate groups compared with the HC group, but the difference between the mild and moderate groups was not significant (Fig. 2E and Fig. S3C). Subsequently, differential abundance analysis was performed. First, the microbial compositions of the HC and CD groups were compared using LinDA. Differential abundance tests revealed that 554 species were differentially abundant between HC and CD groups. Fifty microbiotas were over-represented in HC, whereas 504 microbiotas were over-represented in CD (Fig. 2F, FDR <  = 0.05 and |log2FC|> = 1) (Table S5). Then, TSEA was performed and identified numerous terms related to colorectal cancer and 17 other terms (Fig. 2G and H). Second, in patients with CD, the mild and moderate groups were compared at the species level. Eight bacteria (Fig. 2I, *p* <  = 0.001 and |log2FC|> = 1) were identified (Table S6), but among them, bacteria that did not differ between the HC and CD groups were excluded. Three bacterial species were identified. *Alistipes shahii* and *Pseudodesulfovibrio aespoeensis* were over-represented in the mild group, whereas *Polynucleobacter wuianus* was over-represented in the moderate group (Fig. 2J). Third, a correlation between the CD activity and bacterial abundance was identified (Table S7). The abundance of *A. shahii* (*R* = -0.57, *p* = 1.41E-11) and *P. aespoeensis* (*R* = -0.46, *p* = 1.59E-07) were negatively correlated with CD activity. In contrast, the abundance of *Polynucleobacter wianus* (*R* = 0.35 and *p* = 8.77E-05) showed a positive correlation.

### Alteration in gut microbiome according to the activity of UC

**Fig. 3:**
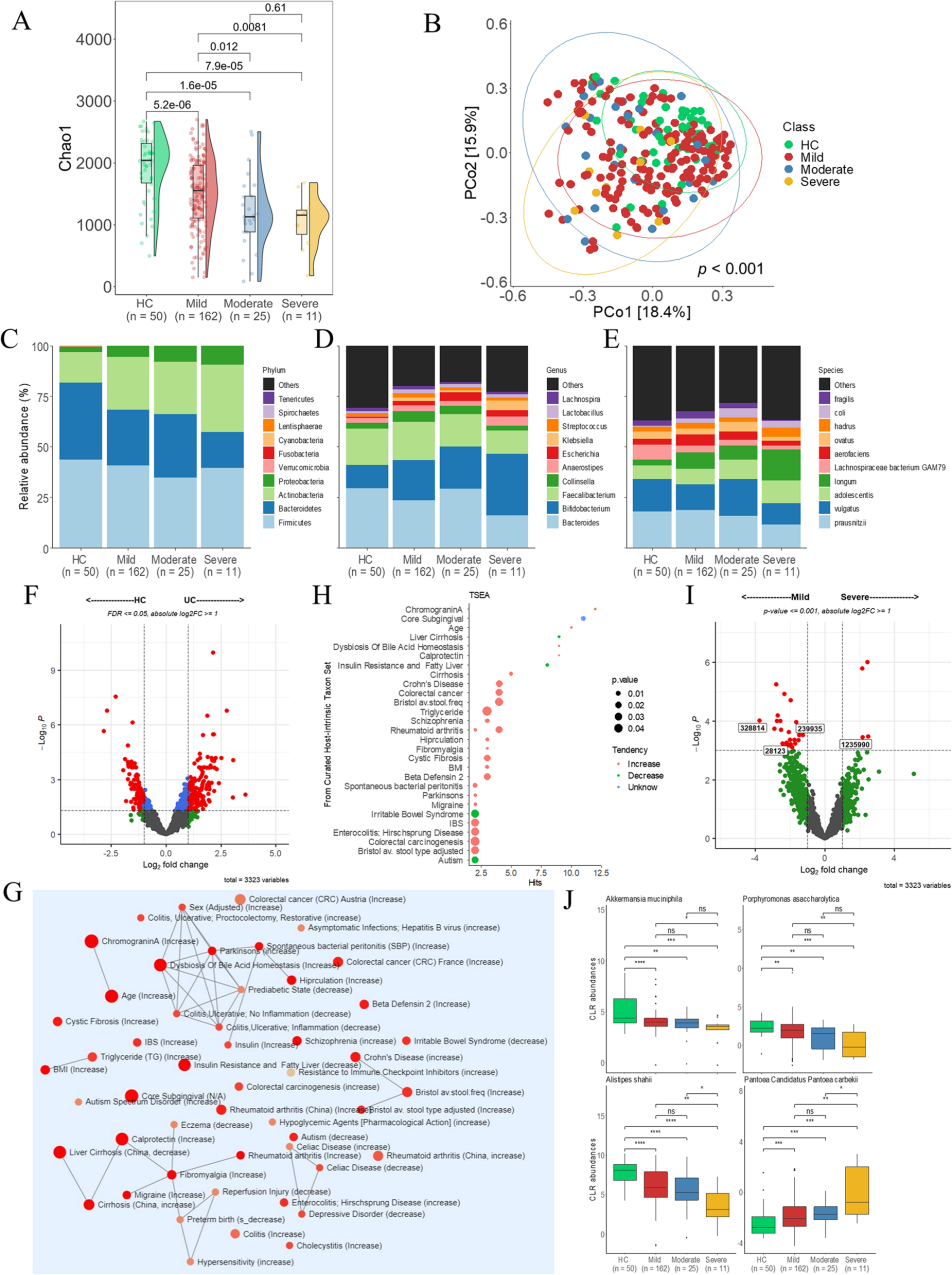
Diversity and distribution of microbiomes in the subgroups of UC according to disease severity. **A** Alpha diversity measured by the Chao1 index. **B** Bray–Curtis-based Beta diversity. Relative abundance of the gut microbiome of the levels of UC activity groups at the phylum (**C**), genus (**D**) and species (**E**) levels. **F** The difference in microbial abundances between HC and UC (FDR <  = 0.05, |log2FC|> = 1). **G** Network modules produced by the microbiota. **H** The microbiota between HC and UC have been implicated in various human diseases (*p* <  = 0.05). **I** Comparison of the microbial composition between the mild and severe groups at the species level (*p* <  = 0.001, log2FC|> = 1). **J** Biomarkers of bacteria associated with the severity of UC. Box plots represent the median and interquartile range. The significances are represented as follows: ***p <  = 0.0001; ***p <  = 0.001; **p <  = 0.01; *p <  = 0.05; and ns, *p* > 0.05

The alpha diversity index decreased in patients with mild, moderate, and severe disease compared to the HC group. Furthermore, the alpha diversity in patients with mild disease differed significantly from those with moderate or severe disease. In contrast, there were no differences in the Chao1 index between the patients with moderate and severe disease. PCoA based on the Bray–Curtis dissimilarity index identified significant differences in microbial composition among the HC, mild, moderate, and severe groups (Fig. 3A and B). The composition of relatively abundant microbiomes differed between the HC and UC groups. Among patients with UC, the mild and moderate groups were similar, but the mild and severe groups differed. The following percentages are in the order of HC > mild > moderate > severe groups. At the phylum level, *Actinobacteria* (15.22, 26.00, 25.97, and 33.16%) increased in the mild and severe groups compared with the HC group. However, *Spirochaetes* (0.03, 0.02, 0.01, and 0.01%) and *Cynobacteria* (0.04, 0.03, 0.02, and 0.02%) decreased (Fig. 3C and Fig. S4A). At the genus level, *Bifidobacterium* (11.42, 19.80, 20.84, and 30.44%) increased in patients with mild, moderate, and severe UC, respectively, compared to HC. However, there were no significant differences between the patients with UC (Fig. 3D and Fig. S4B). At the species level, the abundance of the *Lachnospiraceae bacterium GAM79* (7.53, 3.47, 2.45, and 2.19%) was lower in the UC group than in the HC group, but the difference between the moderate and severe groups was insignificant (Fig. 3E and Fig. S4C). Differential abundance between HC and UC was then compared using LinDA. Between the HC and UC groups, 332 species were differentially abundant. In the HC group, 170 bacteria were over-represented, whereas 162 were over-represented in the UC group (Fig. 3F, FDR < 0.05 and |log2FC|> = 1). (Table S8). These bacteria are associated with many diseases, including irritable bowel syndrome (IBS), colorectal cancer, enterocolitis, and UC (Fig. 3G and H). Second, we compared patients with mild and severe UC at the species level and identified 30 bacterial species. Among them, bacteria that did not change between patients with HC and UC were omitted (Fig. 3I, *p* < 0.001 and |log2FC|> = 1) (Table S9). Therefore, a total of four microbial communities were discovered. *Pantoea Candidatus Pantoea carbekii* increased in the severe group, whereas *Porphyromonas asaccharolytica*, *Akkermansia muciniphila*, and *A. shahii* decreased (Fig. 3J). Third, a correlation between UC disease activity and species abundance was identified (Table S10). UC activity was negatively correlated with the abundance of *A. shahii* (*R* = -0.42, *p* = 2.97E-12), *P. asaccharolytica* (*R* = -0.30, *p* = 9.08E-07), and *A. muciniphila* (*R* = -0.37, *p* = 2.00E-09). However, *P. candidatus Pantoea carbekii* exhibited a positive correlation (*R* = 0.31, *p* = 4.51E-07).

### Classification of IBD by machine learning with microbiome

**Fig. 4:**
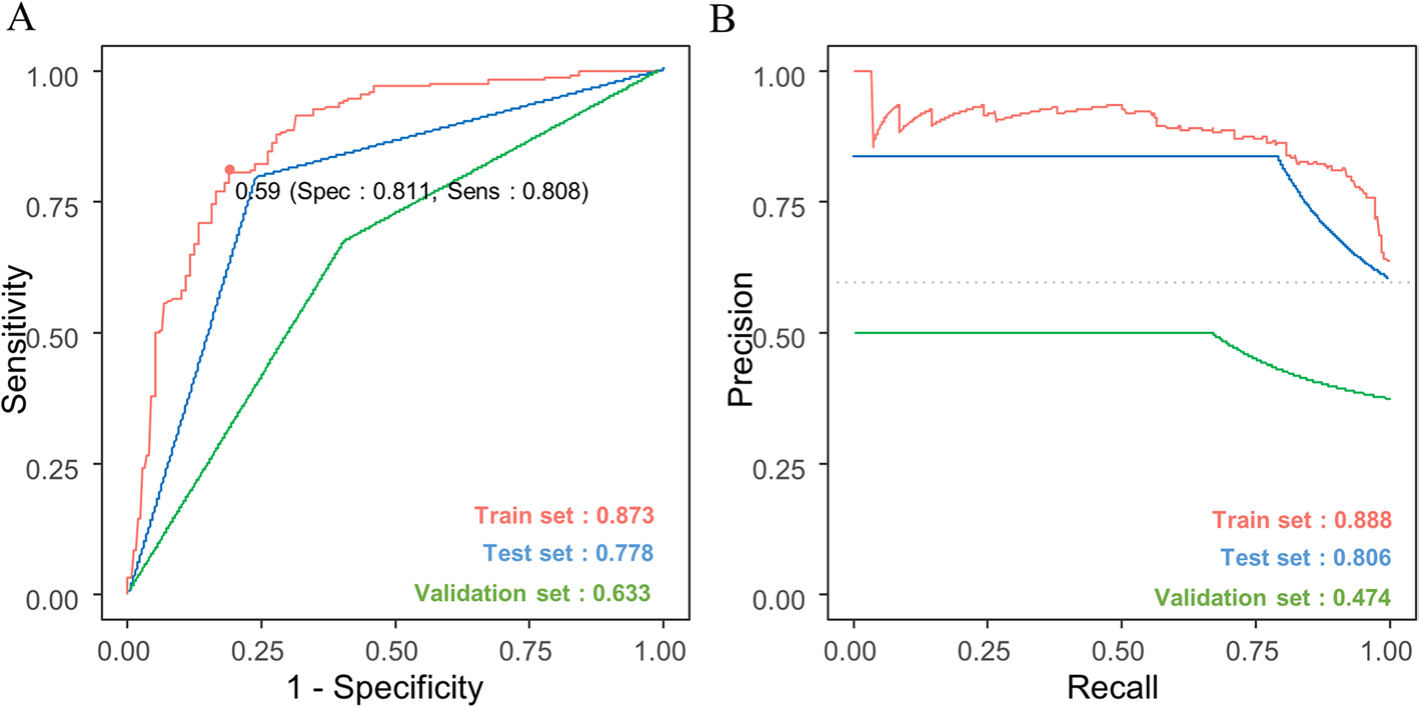
The performance of discriminating CD from UC using 68 microbiome biomarkers using a ridge regression model. **A** Receiver operating characteristic (ROC) curve. **B** Precision-recall (PR) curve

After establishing that CD and UC have different microbial compositions, we attempted to develop a microbiota-based diagnostic model to distinguish between these two diseases. Lazy predict was used to compare classification models to get the best-performing method. Regarding the classification accuracy for CD and UC, ridge logistic regression had the highest accuracy, F1 score and area under the curve (AUC), followed by LinearSVC, RidgeClassifiter, and LinearDiscriminantAnalysis (Fig. S5A, B). Therefore, a penalized (regularized) logistic ridge regression model was used with tenfold cross-validation to select the best method. The optimal lambda (log [λ] = -2.016529) and accompanying regression coefficients were selected as the location where the mean square error (MSE) was the lowest (Table S11 and Fig. S6A). Using the ROC analysis, criterion-related cut-off values were generated to predict IBD. Based on the ideal cut-off threshold (0.59) of the training set, sensitivity and specificity were determined for each data set. We obtained the AUCs of 0.873 and 0.778 for the training and test sets. The model was validated in an independent cohort of 50 IBD patients based on the logistic regression coefficients of the selected predictors. As a result, an AUC of 0.633 was confirmed in the validation cohort (Fig. 4A). PRAUC of 0.888, 0.806 and 0.474 were achieved for the training, test and validation sets (Fig. 4B). In a test set of 128 IBD, the accuracy, sensitivity, specificity, precision, and F1 scores were 0.781, 0.765, 0.792, 0.709, and 0.736. In a validation cohort of 50 IBD, the accuracy, sensitivity, specificity, precision, and F1 scores were 0.625, 0.600, 0.667, 0.750, and 0.667 (Table 4).

**Table 4:** A summary of the diagnostic performance of the microbiome-based biomarker panel in the test set and validation cohorts

Data set	Accuracy	Sensitivity	Specificity	Precision	F1 score
Test set	0.781 95% CI: (0.700, 0.850)	0.765	0.792	0.709	0.736
Validation set	0.625 95% CI: (0.510, 0.730)	0.600	0.667	0.750	0.667

Finally, when we compared the 68 microbes with those in previous studies, we found three overlapping species (*Morganella morganii*, *Faecalibacterium prausnitzii*, and *Haemophilus parainfluenzae*) [17] (Fig. S6B).

## Discussion

In recent decades, considerable progress has been made in the diagnosis and treatment of IBD and our knowledge of the gut microbiome [18]. The gut microbiome contains many potential biomarkers associated with disease activity and treatment efficacy in patients with IBD. Using machine learning classification based on gut microbiome analysis of fecal samples, we provide a non-invasive approach to IBD diagnosis that is practical and effective compared to invasive procedures like colonoscopy.

We identified 68 microbiomes from 3,323 species using LinDA to compare the dominance of each community in CD and UC. Of the 68 microbiomes, 6 species belonged to *Citrobacter*, 4 species belonged to *Escherichia,* and 3 species belonged to *Shigella*. The microbiomes known to cause inflammation were abundant in CD. Especially, the abundance of *E. coli* and *S. dysenteriae* was significantly higher in CD. Adherent invasive *E. coli* can invade and attach to intestinal epithelial cells and proliferate inside macrophages [19]. On the other hand, in UC, anti-inflammatory bacteria were more abundant. Eight species were identified as belonging to *Prevotella* and 2 species belonging to *Comamonas*. *Prevotella,* through its antibacterial effect, can prevent the proliferation of other pathogens, significantly alleviating the severity of IBD [20]. Furthermore, three species of the gut microbiome, *P. mirabilis*, *M. morganii*, and *C. amalonaticus*, showed a significant increase in patients with CD and UC compared to that in HC. This result is consistent with previous literature [21–23]. The metabolic activity of *M. morganii* produces indolimins that cause DNA damage. *M. morganii* increased intestinal permeability and exacerbated colon tumorigenesis in gnotobiotic mice [21].

In previous studies, a machine learning model for IBD diagnosis was trained using information from genomic databases [24], the gut microbiome [25, 26], and histological and endoscopic findings [27]. We applied machine learning methods to the microbiome data to distinguish between CD and UC. Our results showed that the model performed better than previous models. For instance, a previous study in which ML models were trained with data from bacterial genus and operational taxonomic unit (OTU) data from only 20 patients with CD and 19 with UC, achieved 0.79 AUC and 0.72 AUC, respectively [28]. Our study achieved predictive performance with 0.873 AUC in the train set and 0.778 AUC in the test set. Additionally, when we validated our model using an independent cohort, we attained performance of an AUC of 0.633. We suggest that the AUC of 0.633 in an independent cohort is reasonably good, considering the differences in cohorts (Korean vs. American) and sequencing platforms (MGI vs. Illumina), which may cause batch effects.

We identified patterns in which some bacteria changed continuously as the disease progressed in patients with CD and UC. This result suggests that assessing the severity of a disease utilizing specific microbial biomarkers (anti- and pro-inflammatory bacteria) may help predict and monitor the effectiveness of an intervention [29]. By analyzing their association with CD activity, *A. shahii*, *P. aespoeensis* and *P. wuianus* were identified. In contrast, the microbiota associated with disease activity in UC includes *P. candidatus pantoea carbekii*, *A. shahii*, *A. muciniphila*, and *P. asaccharolytica*. Interestingly, *A. shahii* was common in both diseases. The depletion of *Alistipes* is an important indicator of a gut microbiome imbalance, although *A. shahii* has a less clearly defined metabolic role in the gut microbiota [30]. Recent research has highlighted the significant function of *A. shahii* as an inhibitory regulator of tumor development and its protective activity against various disorders [31, 32]. Like *A. shahii*, *A. muciniphila* acts as an immunomodulator in the intestine through regulatory T cells that produce IL-10 [33]. *A. muciniphila* reduces inflammatory cytokines and chemokines (TNF-, IL1-, IL6, IL12A, MIP-1A, G-CSF, and KC) in the serum and tissues [34]. The known colitis-causing bacteria *Clostridium difficile*, *Ruminococcus gnavus*, *Bacteroides fragilis* and *E. coli,* were not associated with disease activity in any of the diseases.

Most previous studies on various gut microbiomes, including IBD, have used 16S ribosomal RNA gene sequencing, which can accurately identify microbiomes at the phylum level but is limited at the species level [35]. In contrast, the whole-metagenome shotgun (WMS) sequencing method allows taxonomy to be defined more accurately at the species level and identify specific genes [36, 37]. Our study is more accurate than previous studies in identifying and comparing species levels using WMS sequencing. Another strength was the large sample size. IBD research using WMS technology has been carried out on a small scale, but our study used a large sample size, resulting in a better performance of the diagnostic model.

The present study had a few limitations. First, confounders, including BMI, age, drug use, lifestyle, and enterotypes, were not controlled for, which may affect the microbiome. Second, while we validated our model in an independent cohort of 80 Western patients, another validation in a larger cohort will strengthen the usefulness of our microbiome markers. Nonetheless, identifying fecal microbiomes associated with disease phenotypes and activity at the species level will advance our knowledge in the field.

## Conclusions

CD and UC had significant differences in microbial diversity and gut microbial community composition. We classified the two diseases using shotgun metagenomics and machine learning approaches. A potential biomarker for predicting disease progression has been proposed by comparing the activity of each disease. These analyses could aid in developing novel prognostic and therapeutic strategies for CD and UC using the gut microbiome.

## Materials and methods

### Data collection

Two hospitals (Chungnam National University Hospital and Daejeon St. Mary’s Hospital) participated in this study. The biospecimen and data used in this study were provided by the Biobank of Chungnam National University Hospital, a member of the Korea Biobank Network. We collected the clinical data from newly diagnosed and monitored patients with CD and UC). Patients with CD and UC were divided into three subgroups according to disease activity (mild, moderate, or severe). To characterize the microbiome associated with disease severity in each group, we excluded patients who used drugs that could affect the gut microbiome and those who underwent disease-related surgery.

### Public data processing

Metagenomics data and clinical data generated through the Inflammatory Bowel Disease Multiomics Database website (IBDMDB http://ibdmdb.org) were downloaded from the Sequence Read Archive (SRA). Among them, the Shotgun metagenome sequencing data was downloaded by default, and the microbial abundance file was used as an alternative to the original Shotgun metagenome sequencing fastq file. If the host_id was the same, samples from the initial fecal collection date were used. Thus, CD (*n* = 50) and UC (*n* = 30) were used to validate the machine learning model. MBECS (https://github.com/rmolbrich/MBECS) was used to correct for the batch effect between the two studies.

### DNA shotgun sequencing

Fecal samples were collected between 2019 and 2020, and DNA was extracted from the fecal sample and stored at -80 °C until use. gDNA (1 mg) was sheared using an S220 Ultra Sonicator (Covaris, Woburn, MA, USA). Library preparation was performed using the MGIEasy DNA Library Prep Kit (MGI, China), according to the manufacturer’s instructions. Briefly, after size selection of the fragmented gDNA using AMPure XP magnetic beads, the fragmented gDNA was end-repaired and A-tailed at 37 °C for 30 min and 65 °C for 15 min. The index adapter was ligated to the ends of the DNA fragments at 23 °C for 60 min. After the adapter-ligated DNA was removed, PCR was performed to enrich the DNA fragments with adapter molecules. Thermocycler conditions were as follows: 95 °C for 3 min, 7 cycles at 98 °C for 20 s, 60 °C for 15 s, and 72 °C for 30 s, with a final extension at 72 °C for 10 min. The double-stranded library was quantified using the QauntiFluor ONE dsDNA system (Promega, Madison, WI, USA). The library was circularized at 37 °C for 30 min and then digested at 37 °C for 30 min, followed by cleanup of the circularization product. The library was incubated at 30 °C for 25 min using the DNA nanoball (DNB) enzyme to make the DNB. Finally, the library was quantified using the QuantiFluor ssDNA System (Promega). The prepared DNB was sequenced on the MGIseq system (MGI) with 150 bp paired end reads at a single run.

### Processing of sequence data

Quality control of the raw sequences of each sample was performed using the FastQC software (ver. 0.11.9). Primer sequences were removed using Cutadapt, and low-quality sequences were trimmed using Trim Galore (ver. 0.6.4). Human host reads were subtracted by mapping them to the human reference genome (GRCh38) using the Burrows-Wheeler Aligner (BWA) (ver. 0.7.17). After mapping, the FASTQ-paired files were sorted using Samtools Sort (ver. 1.10). Taxonomic classification was performed using the standard taxonomic sequence classification tool Kraken2 (ver. 1.1.1), and relative abundance was estimated using Bayesian Reestimation of Abundance with KrakEN (Bracken) (ver. 2.6.2). After removing low-abundance reads, the data was normalized in several ways (rarefaction and centered log ratio) using R (ver. 4.1.1). Based on the MetaCyc database, HMP Unified Metabolic Analysis Network 3 (HUMAnN3) (ver. 3.0.0) was used to describe the metabolic potential of individuals within a microbial population. The data were then combined using a join table script. An unstratified table was obtained using the split_stratified_table script. LinDA was used to compare variations in the MetaCyc pathway abundance. The regrouped_table script was used to translate the gene table to uniref90_ko. The KO term was renamed using the rename_table script. Then, using the same procedure as above, an unstratified table was created, and the abundances of KO were compared.

### Enterotypes identification using sample clustering between healthy control and IBD individuals

The enterotype classification was performed using the genus of relative abundance table based on the Jensen-Shannon branch clustering of samples published by the MetaHIT consortium in 2011 in the enterotyping pipeline (https://enterotype.embl.de/#) [38]. Clustering results were visualized in Principal Coordinates Analysis (PCoA) plots using the ade4 package.

### Statistical analysis of metagenome data

R software (ver. 4.1.1) was used for statistical analyses and visualization. Chi-square and Fisher’s exact tests were used to assess the association between clinical characteristics and microbiome composition. The Wilcoxon rank-sum test and Student’s t-test were used to evaluate the significance of differences in microbiomes between groups. The abundance table was rarefied to 1,222,561 sequences per sample by random subsampling to analyze microbial diversity in R. Permutation analysis of variance (PERM3ANOVA) was performed using the adonis2 function of the vegan package in R (ver. 4.1.3). LinDA were used to identify significant differences in relative abundance between the two groups at the species level [39]. The analysis codes are provided at https://github.com/kangdy9801/Diagnosis-of-Crohn-s-Disease-and-Ulcerative-Colitis-Using-the-Microbiome.

From a given list of taxa of interest, TSEA examines whether any patterns exist that are biologically or ecologically significant. Each node represents a taxon set with color based on the *p*-value and size based on the number of hits. An edge connects two taxa if the shared hits are > 20% of the combined taxa [40]. Spearman's rank correlation analysis was used to evaluate the correlation between microbiota and disease activity.

### Supervised machine learning: Classification

Utilizing Lazy Predict (https://github.com/shankarpandala/lazypredict), 27 classification models were trained to diagnose diseases based on the microbiota identified by differential abundance analysis. All hyperparameters were set to default. Samples were divided at random into 70% and 30%. The model was trained on 70% of the total sample, and the remaining 30% comprised the test set. The reported results used a ridge logistic regression model and performed the best. After selecting the most optimal model, the performance of the supervised machine learning model utilizing only the training samples was evaluated during the training phase using tenfold cross-validation. Data shuffling and splitting were performed in ten independent iterations. ROC curve analysis was performed to establish the ideal cut-off threshold, sensitivity, and specificity. To overcome the class imbalance problem, we conducted the precision-recall (PR) curve [41, 42]. Seven performance indicators, including accuracy, sensitivity, specificity, precision, AUC, PRAUC and F1 score, were used to assess the models’ performance when tested on the dataset.

### Supplementary Information

**Additional file 1: Fig. S1.** Enterotypes identified in individuals with HC and IBD. (A) Using principal coordinate analysis (PCoA), participants were divided into three enterotypes, with *Bacteroides, Faecalibacterium *or *Bifidobacterium* as the primary distinguishing factors. (B) Three bacteria most abundant among the three enterotypes (Wilcoxon test, *p *< 0.05). **Fig. S2.** Comparison of the relative abundance of microbiota among HC, CD, and UC in phylum (A), genus (B) and species (C) based on the Kruskal-Wallis test. Each figure is shown in the order of HC, CD and UC. **Fig. S3.** Comparison of the relative abundance of microbiota between disease stages (HC, mild, and moderate) in CD patients at the phylum (A), genus (B) and species (C) levels. The numbers are listed in the following order: HC, mild and moderate. **Fig. S4.** Comparison of the relative abundance of microbiota between disease stages in UC patients at the levels of phylum (A), genus (B), and species (C) levels. The numbers are listed in the following order: HC, mild, moderate, and severe. **Fig. S5.** Comparison of 27 supervised machine learning models for diagnosing IBD subtypes using differential abundance. (A) F1 score; (B) ROC AUC. **Fig. S6.** (A) Parameter adjustment of a supervised machine learning model to classify CD and UC individuals using gut microbiomes. Ten-fold cross-validation was performed to select the optimal lambda. The point where the mean square error (MSE) was minimized was designated as the best lambda (log [λ] = -2.016529). (B) Venn diagram comparing our microbiome list with that of Clonney et al.. **Fig. S7.** Graphical abstract

**Additional file 2: Supplementary Table 1.**  Association between enterotypes and IBD occurrence.

**Additional file 3: Supplementary Table 2.** Differences in the taxonomic composition of the gut microbiome in patients with CD and UC. **Supplementary Table 3.** Metacyc pathway of the different gut microbiomes (CD VS UC). **Supplementary Table 4.** KEGG Orthology of the different gut microbiomes (CD VS UC). **Supplementary Table 5.** Differences in the taxonomic composition of the gut microbiome in HC and CD. **Supplementary Table 6.** Differences in the taxonomic composition of the gut microbiome in mild and Moderate patients with CD. **Supplementary Table 7.** Correlation between microbes and severity of CD. **Supplementary Table 8.** Differences in the taxonomic composition of the gut microbiome in HC and UC. **Supplementary Table 9.** Differences in the taxonomic composition of the gut microbiome in mild and Severe patients with UC. **Supplementary Table 10.** Correlation between microbes and the severity of UC. **Supplementary Table 11.** Microbial coefficients used for modeling

## Data Availability

The data sets utilized in this research are available from NCBI’s Sequence Read Archive (SRA) (Accession ID: PRJNA945504) and Korean Nucleotide Archive (KoNA) (Accession ID: PRJKA220304) (https://www.kobic.re.kr/kona/).
