# Supplementary material for: Diagnosis of Crohn’s disease and ulcerative colitis using the microbiome
Source: BMC Microbiol. 2023 Nov 11;23:336. doi: 10.1186/s12866-023-03084-5 (PMC10640746; doi:10.1186/s12866-023-03084-5)
Supplement: Supplementary file 1 — Additional file 1: Fig. S1. Enterotypes identified in individuals with HC and IBD. (A) Using principal coordinate analysis (PCoA), participants were divided into three enterotypes, with Bacteroides, Faecalibacterium or Bifidobacterium as the primary distinguishing factors. (B) Three bacteria most abundant among the three enterotypes (Wilcoxon test, p < 0.05). Fig. S2. Comparison of the relative abundance of microbiota among HC, CD, and UC in phylum (A), genus (B) and species (C) based on the Kruskal-Wallis test. Each figure is shown in the order of HC, CD and UC. Fig. S3. Comparison of the relative abundance of microbiota between disease stages (HC, mild, and moderate) in CD patients at the phylum (A), genus (B) and species (C) levels. The numbers are listed in the following order: HC, mild and moderate. Fig. S4. Comparison of the relative abundance of microbiota between disease stages in UC patients at the levels of phylum (A), genus (B), and species (C) levels. The numbers are listed in the following order: HC, mild, moderate, and severe. Fig. S5. Comparison of 27 supervised machine learning models for diagnosing IBD subtypes using differential abundance. (A) F1 score; (B) ROC AUC. Fig. S6. (A) Parameter adjustment of a supervised machine learning model to classify CD and UC individuals using gut microbiomes. Ten-fold cross-validation was performed to select the optimal lambda. The point where the mean square error (MSE) was minimized was designated as the best lambda (log [λ] = -2.016529). (B) Venn diagram comparing our microbiome list with that of Clonney et al.. Fig. S7. Graphical abstract [file 12866_2023_3084_MOESM1_ESM.pdf]

Supplementary file

Diagnosis of Crohn's Disease and Ulcerative Colitis Using the Microbiome

Da-Yeon Kang[1,2]*, Jong-Lyul Park[3]*, Min-Kyung Yeo[4]*, Sang-Bum Kang[5], Jin-Man Kim[4], Ju Seok Kim[6]^, and Seon-Young Kim[7]^

Fig. S1

A

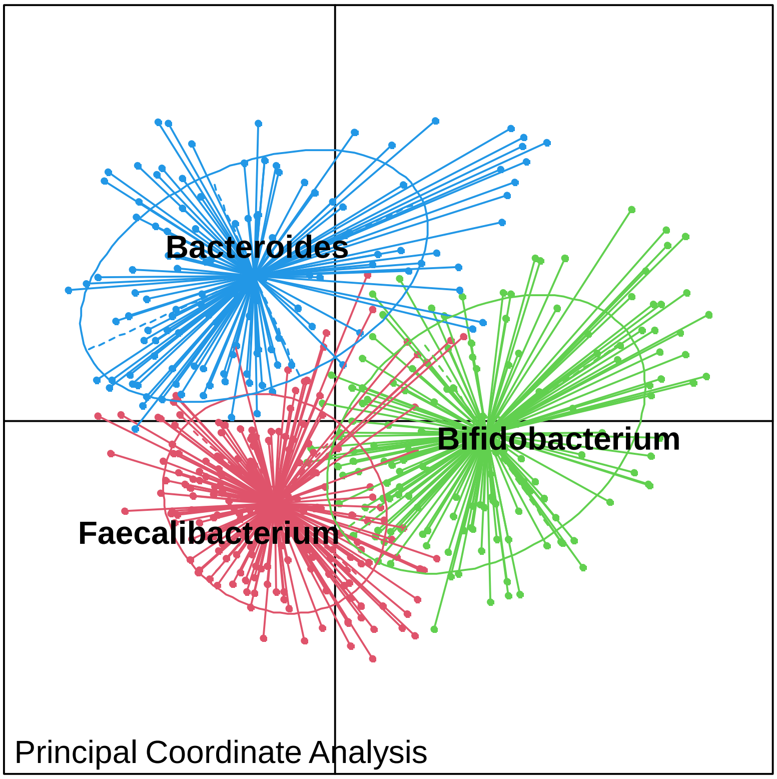
Bacteroides
Bifidobacterium
Faecalibacterium
Principal Coordinate Analysis

B

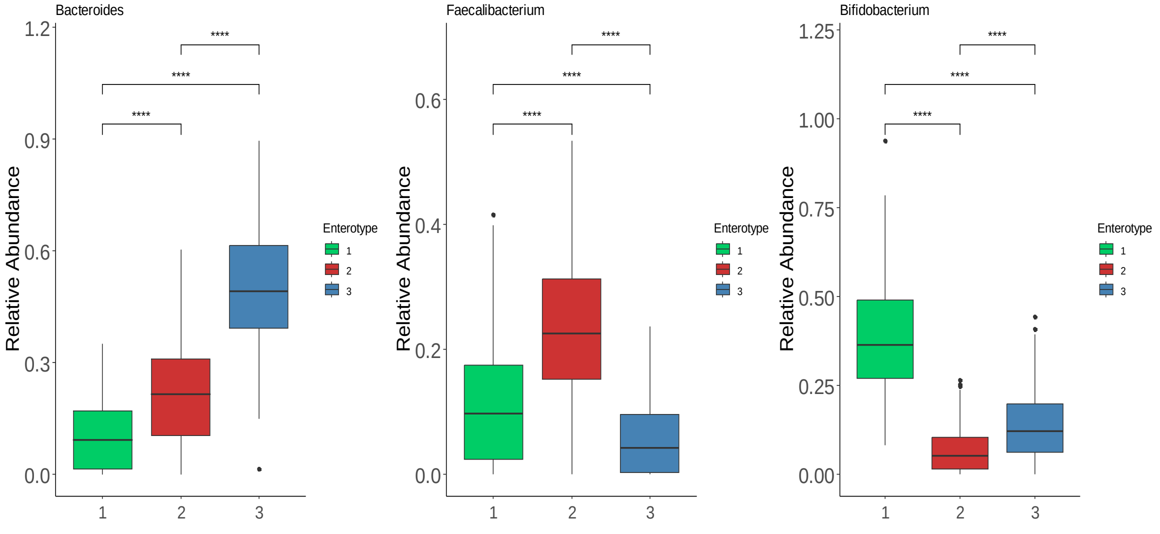
Bacteroides
Faecalibacterium
Bifidobacterium
Relative Abundance
Enterotype
1
2
3
****

Fig. S2

A

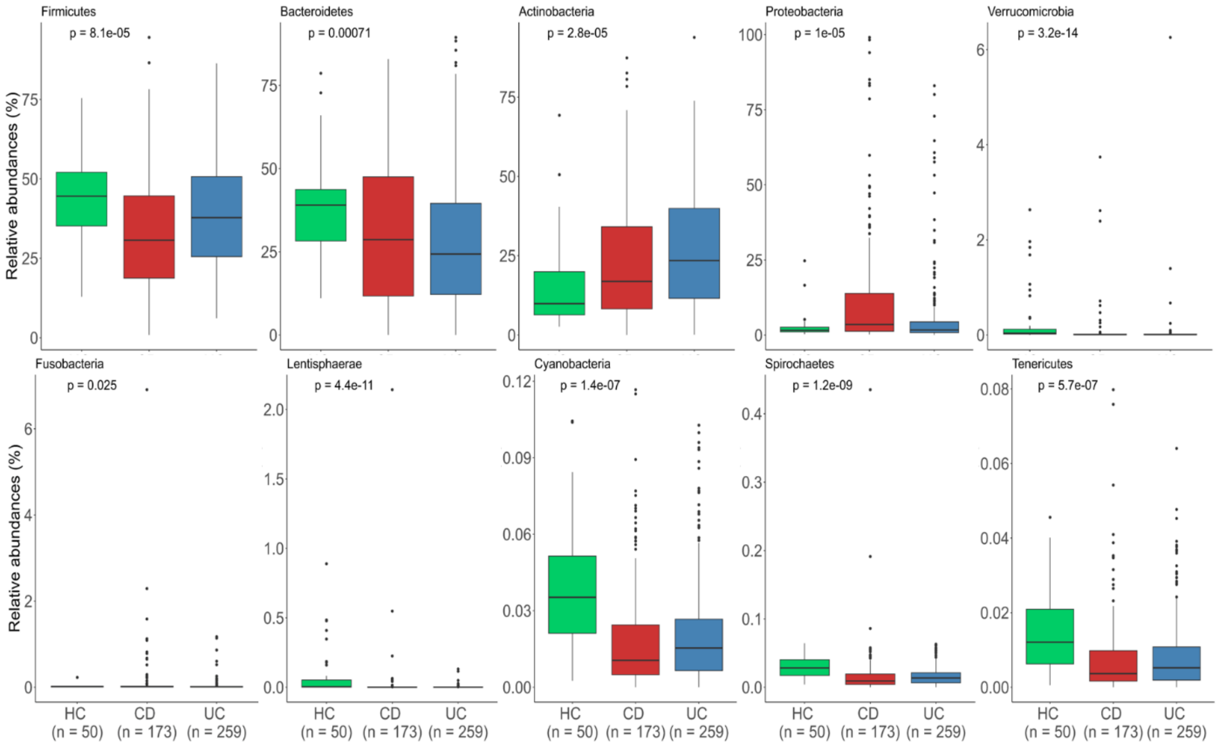
Firmicutes
p = 8.1e-05
Bacteroidetes
p = 0.00071
Actinobacteria
p = 2.8e-05
Proteobacteria
p = 1e-05
Verrucomicrobia
p = 3.2e-14
Fusobacteria
p = 0.025
Lentisphaerae
p = 4.4e-11
Cyanobacteria
p = 1.4e-07
Spirochaetes
p = 1.2e-09
Tenericutes
p = 5.7e-07
Relative abundances (%)
HC (n = 50)
CD (n = 173)
UC (n = 259)

B

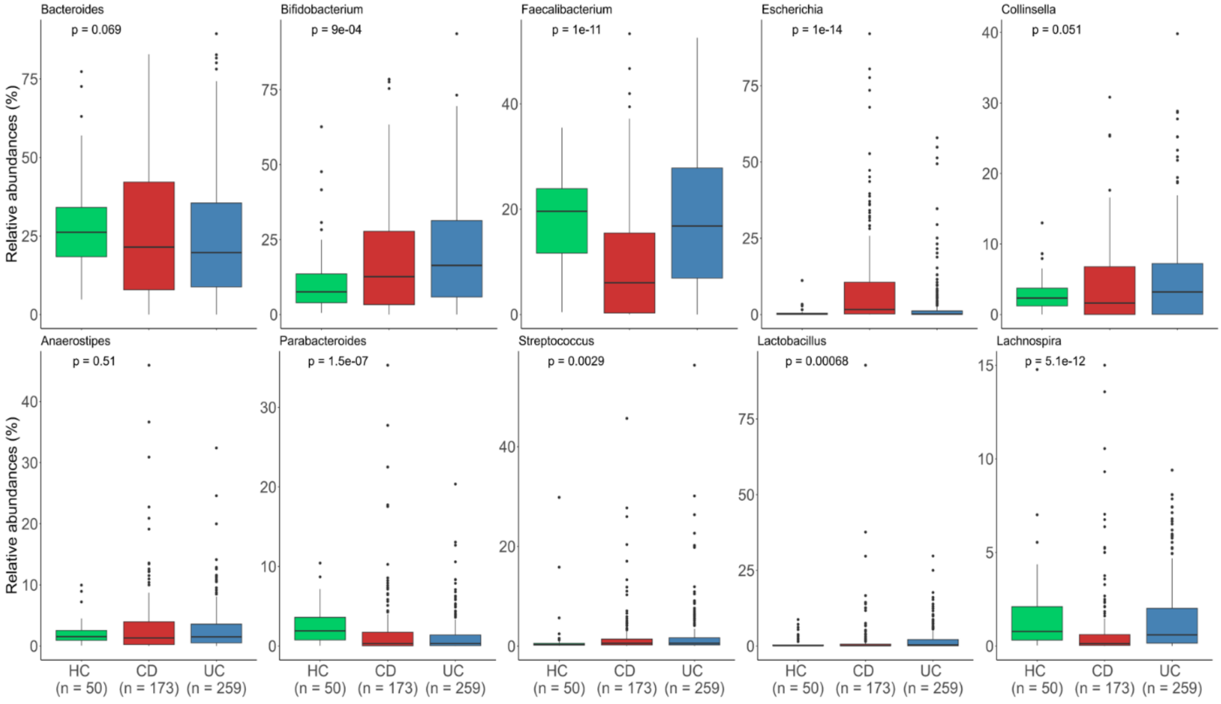
Bacteroides
p = 0.069
Bifidobacterium
p = 9e-04
Faecalibacterium
p = 1e-11
Escherichia
p = 1e-14
Collinsella
p = 0.051
Anaerostipes
p = 0.51
Parabacteroides
p = 1.5e-07
Streptococcus
p = 0.0029
Lactobacillus
p = 0.00068
Lachnospira
p = 5.1e-12
Relative abundances (%)
HC (n = 50)
CD (n = 173)
UC (n = 259)

C

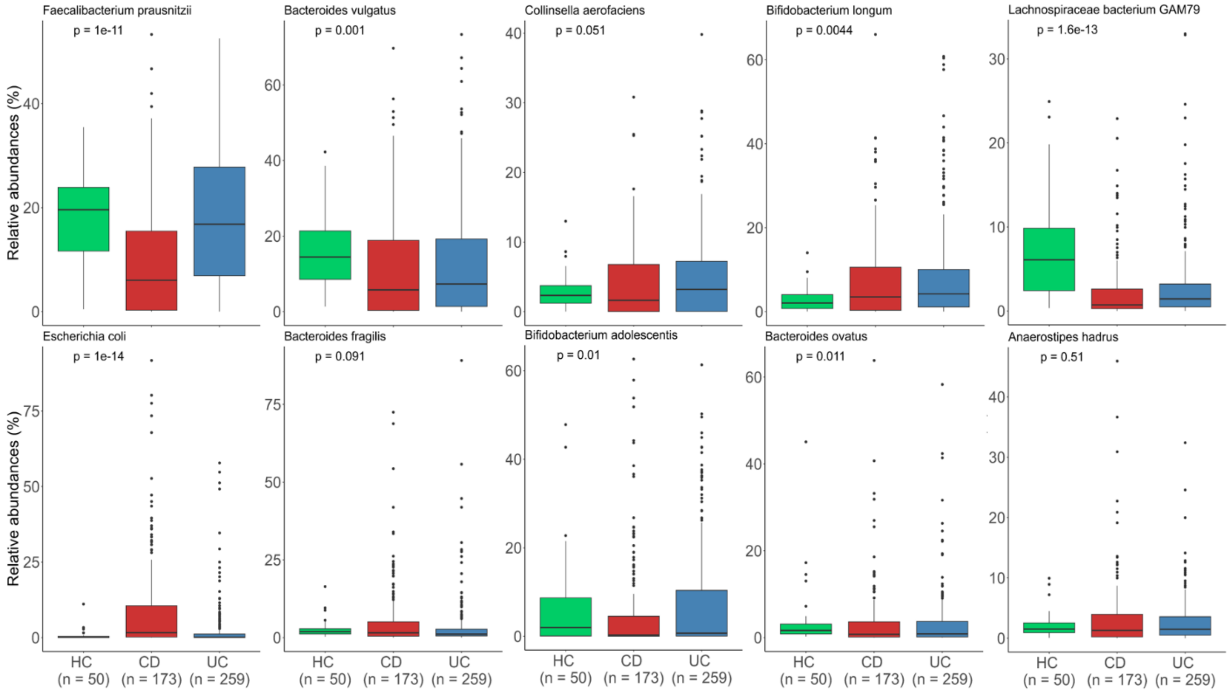
Faecalibacterium prausnitzii
p = 1e-11
Bacteroides vulgatus
p = 0.001
Collinsella aerofaciens
p = 0.051
Bifidobacterium longum
p = 0.0044
Lachnospiraceae bacterium GAM79
p = 1.6e-13
Escherichia coli
p = 1e-14
Bacteroides fragilis
p = 0.091
Bifidobacterium adolescentis
p = 0.01
Bacteroides ovatus
p = 0.011
Anaerostipes hadrus
p = 0.51
Relative abundances (%)
HC (n = 50)
CD (n = 173)
UC (n = 259)

Fig. S3

A

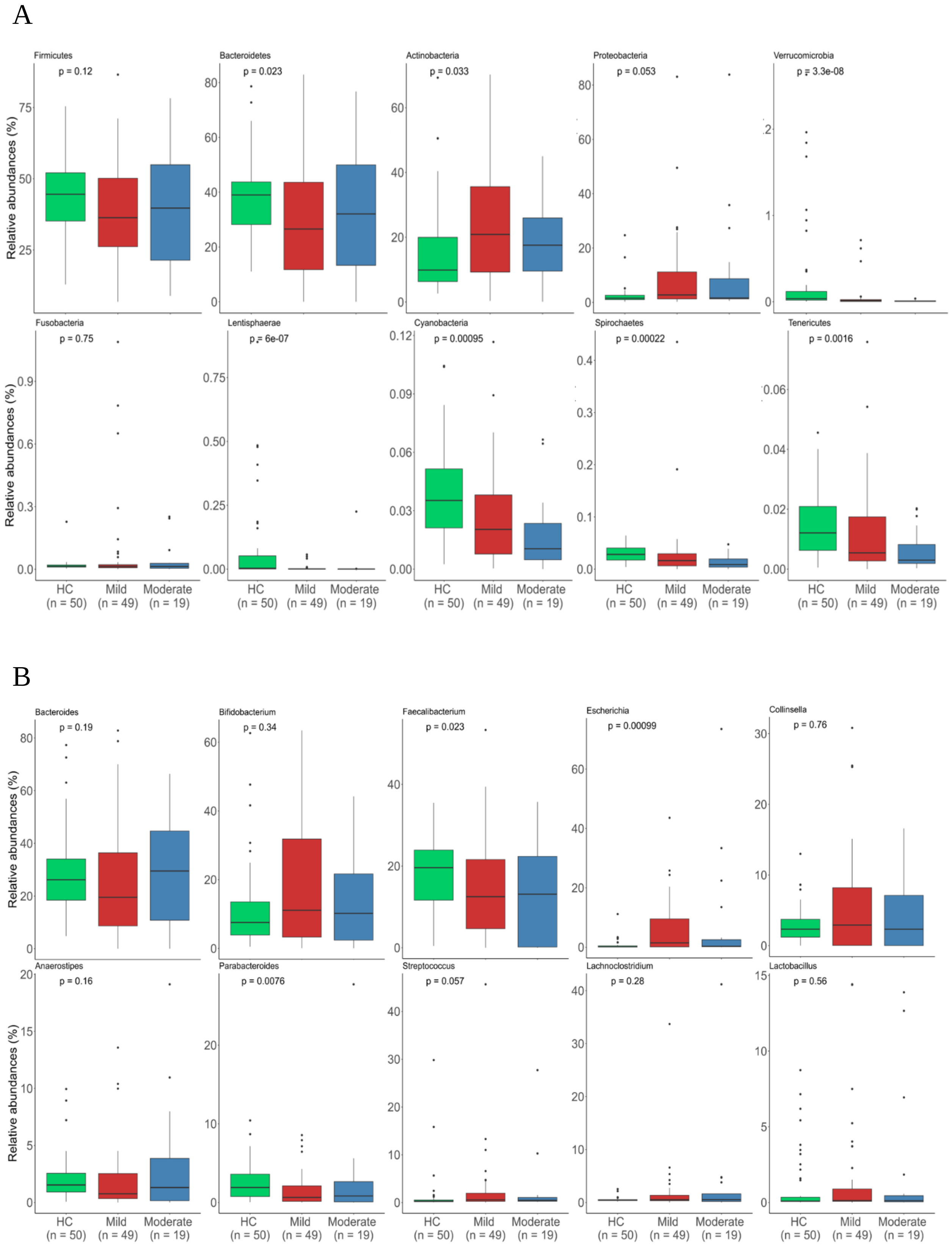

Firmicutes
p = 0.12
Bacteroidetes
p = 0.023
Actinobacteria
p = 0.033
Proteobacteria
p = 0.053
Verrucomicrobia
p = 3.3e-08
Fusobacteria
p = 0.75
Lentisphaerae
p = 6e-07
Cyanobacteria
p = 0.00095
Spirochaetes
p = 0.00022
Tenericutes
p = 0.0016
Relative abundances (%)
HC
(n = 50)
Mild
(n = 49)
Moderate
(n = 19)
B
Bacteroides
p = 0.19
Bifidobacterium
p = 0.34
Faecalibacterium
p = 0.023
Escherichia
p = 0.00099
Collinsella
p = 0.76
Anaerostipes
p = 0.16
Parabacteroides
p = 0.0076
Streptococcus
p = 0.057
Lachnoclostridium
p = 0.28
Lactobacillus
p = 0.56

C

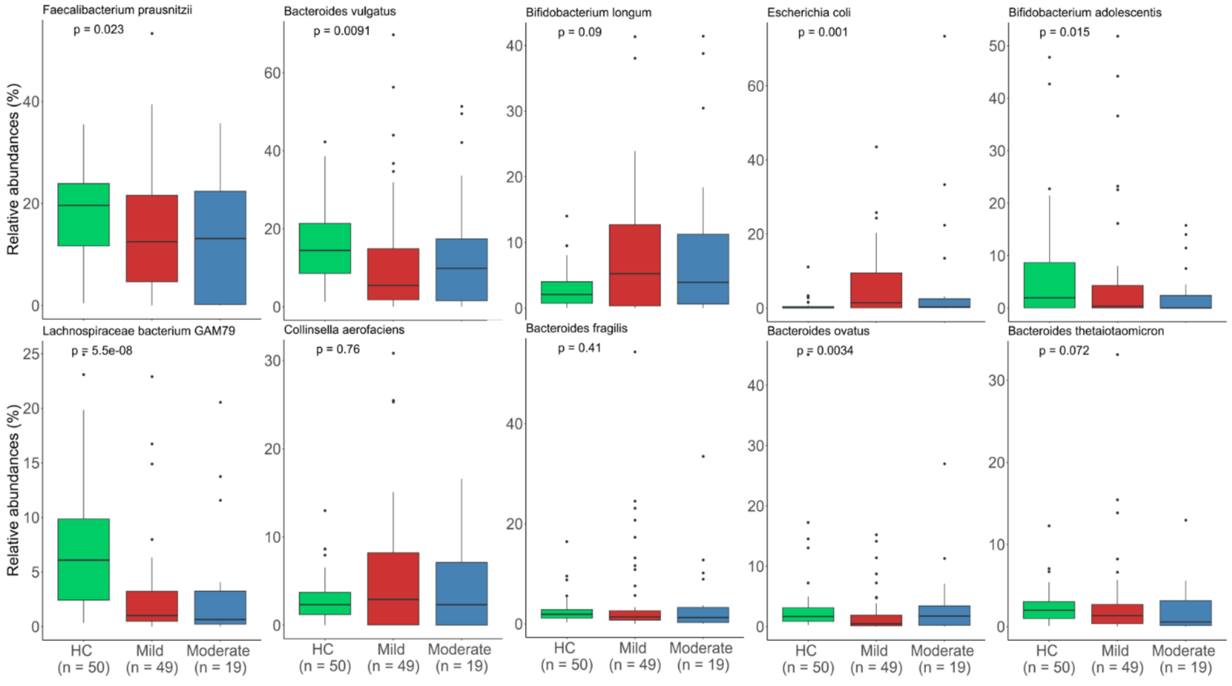
Faecalibacterium prausnitzii
p = 0.023
Bacteroides vulgatus
p = 0.0091
Bifidobacterium longum
p = 0.09
Escherichia coli
p = 0.001
Bifidobacterium adolescentis
p = 0.015
Lachnospiraceae bacterium GAM79
p = 5.5e-08
Collinsella aerofaciens
p = 0.76
Bacteroides fragilis
p = 0.41
Bacteroides ovatus
p = 0.0034
Bacteroides thetaiotaomicron
p = 0.072
Relative abundances (%)
HC (n = 50)
Mild (n = 49)
Moderate (n = 19)

Fig. S4

A

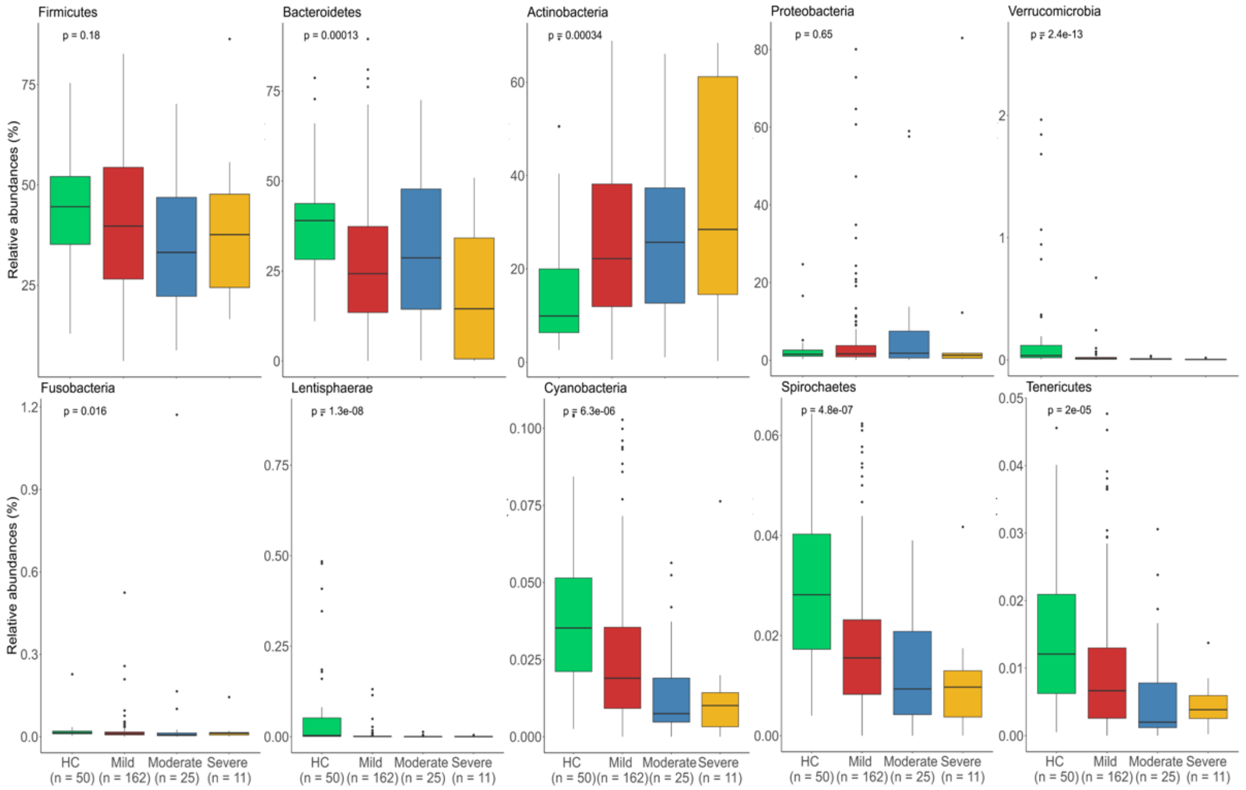
Firmicutes
p = 0.18
Bacteroidetes
p = 0.00013
Actinobacteria
p = 0.00034
Proteobacteria
p = 0.65
Verrucomicrobia
p = 2.4e-13
Fusobacteria
p = 0.016
Lentisphaerae
p = 1.3e-08
Cyanobacteria
p = 6.3e-06
Spirochaetes
p = 4.8e-07
Tenericutes
p = 2e-05
Relative abundances (%)
HC (n = 50)
Mild (n = 162)
Moderate (n = 25)
Severe (n = 11)

B

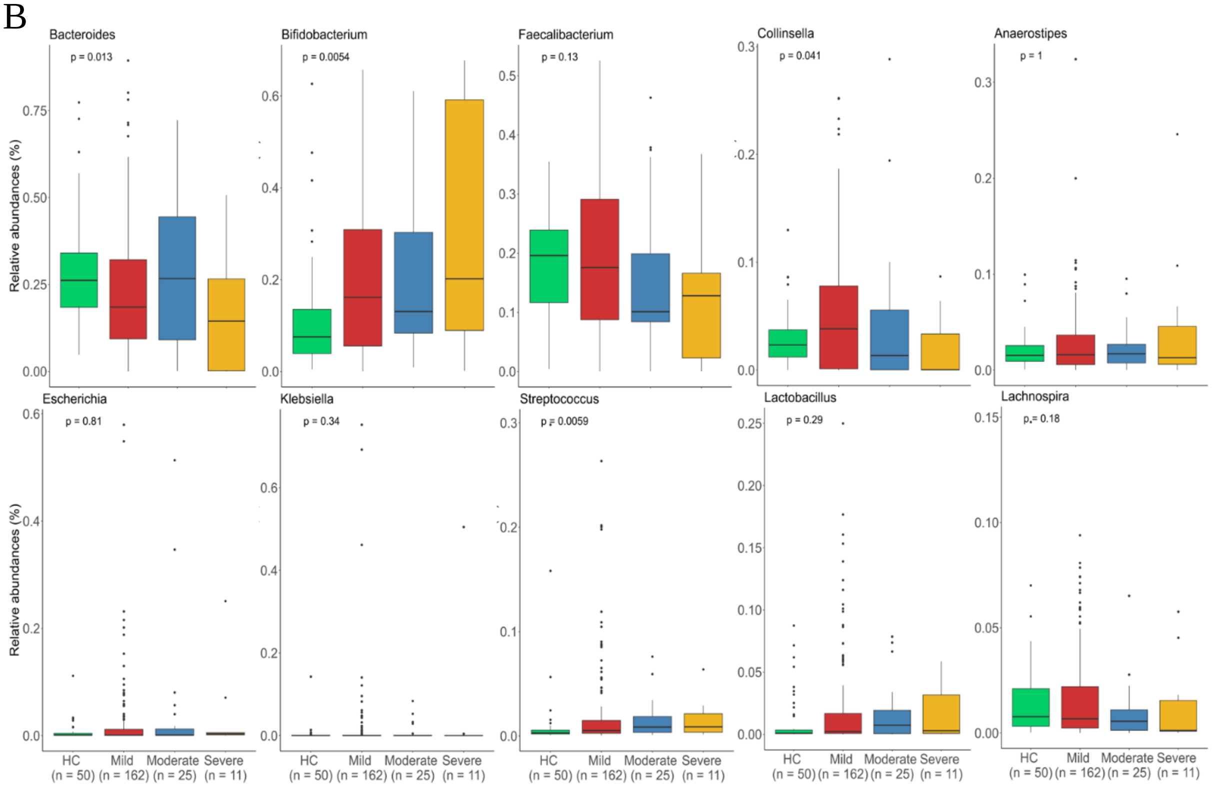
Bacteroides
p = 0.013
Bifidobacterium
p = 0.0054
Faecalibacterium
p = 0.13
Collinsella
p = 0.041
Anaerostipes
p = 1
Escherichia
p = 0.81
Klebsiella
p = 0.34
Streptococcus
p = 0.0059
Lactobacillus
p = 0.29
Lachnospira
p = 0.18
Relative abundances (%)
HC (n = 50)
Mild (n = 162)
Moderate (n = 25)
Severe (n = 11)

C

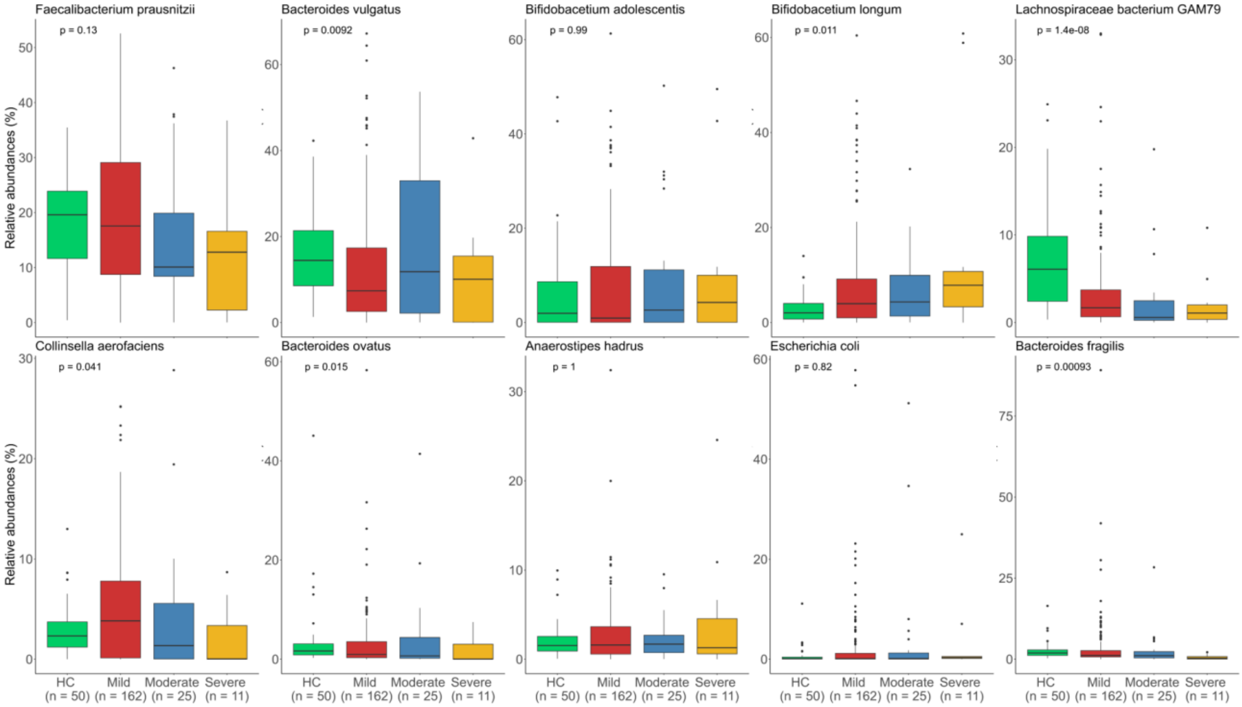

Faecalibacterium prausnitzii
p = 0.13
Bacteroides vulgatus
p = 0.0092
Bifidobacetium adolescentis
p = 0.99
Bifidobacetium longum
p = 0.011
Lachnospiraceae bacterium GAM79
p = 1.4e-08
Collinsella aerofaciens
p = 0.041
Bacteroides ovatus
p = 0.015
Anaerostipes hadrus
p = 1
Escherichia coli
p = 0.82
Bacteroides fragilis
p = 0.00093
Relative abundances (%)
HC (n = 50)
Mild (n = 162)
Moderate (n = 25)
Severe (n = 11)

Fig. S5

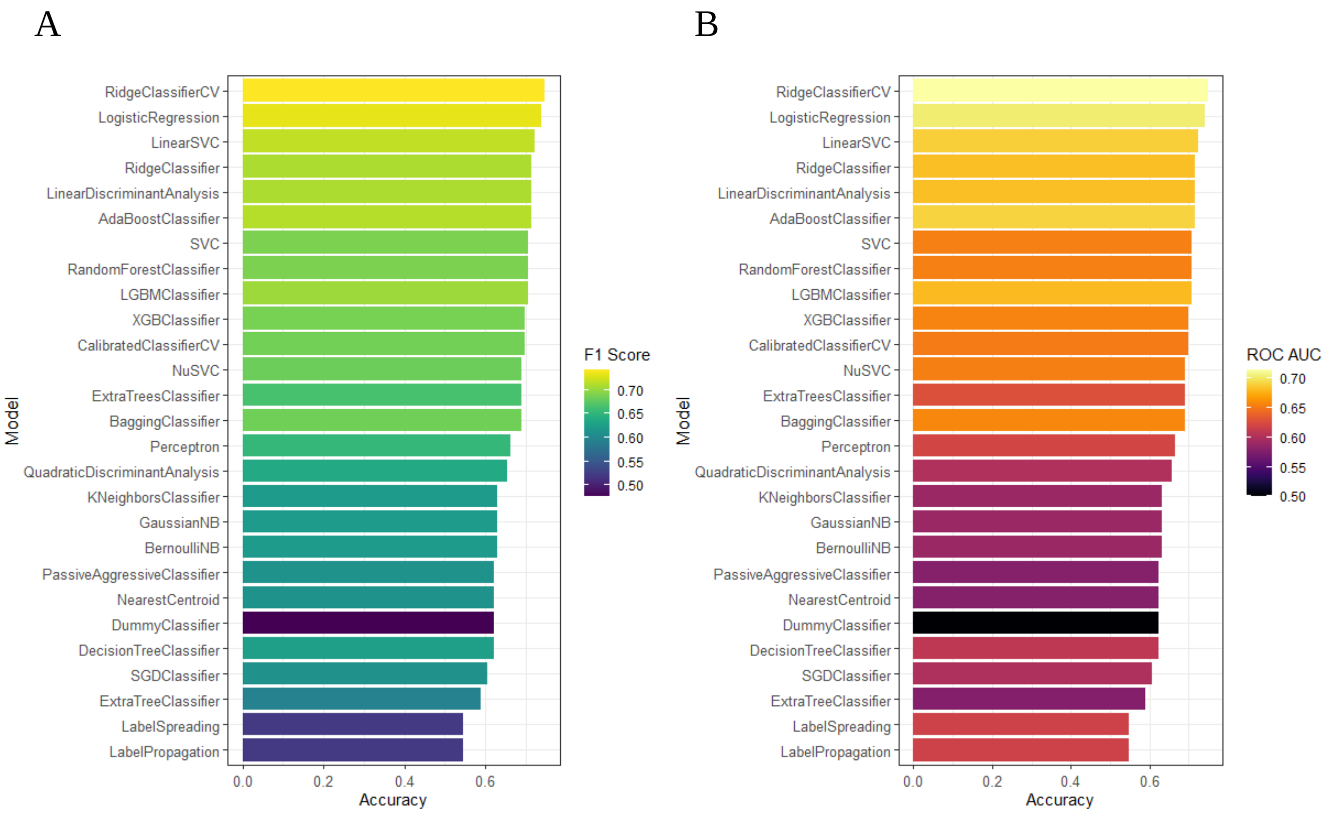
A
B
Model
Accuracy
F1 Score
ROC AUC
RidgeClassifierCV
LogisticRegression
LinearSVC
RidgeClassifier
LinearDiscriminantAnalysis
AdaBoostClassifier
SVC
RandomForestClassifier
LGBMClassifier
XGBClassifier
CalibratedClassifierCV
NuSVC
ExtraTreesClassifier
BaggingClassifier
Perceptron
QuadraticDiscriminantAnalysis
KNeighborsClassifier
GaussianNB
BernoulliNB
PassiveAggressiveClassifier
NearestCentroid
DummyClassifier
DecisionTreeClassifier
SGDClassifier
ExtraTreeClassifier
LabelSpreading
LabelPropagation
0.0
0.2
0.4
0.6
0.70
0.65
0.60
0.55
0.50

Fig. S6

A

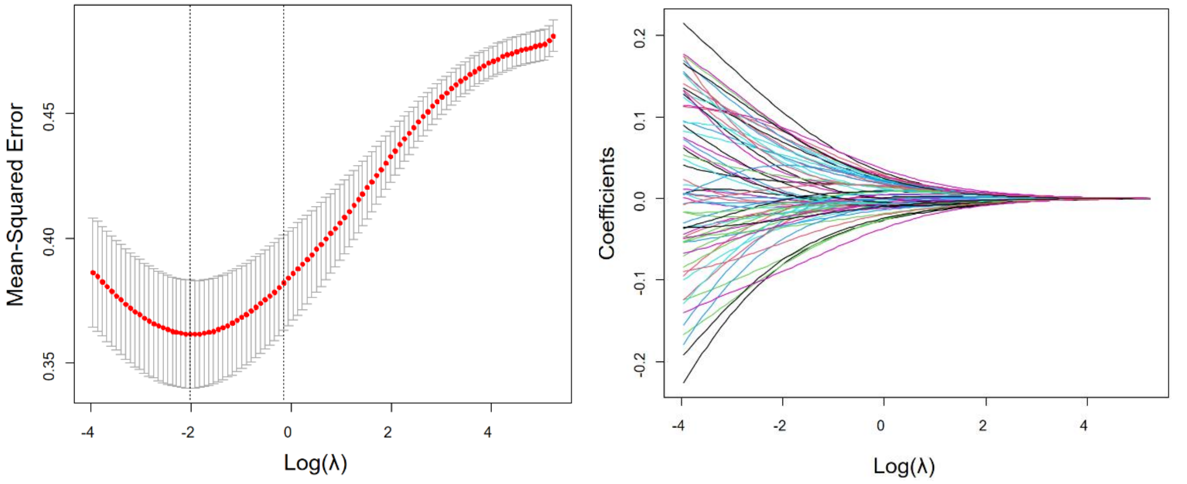

Mean-Squared Error
0.35
0.40
0.45
-4
-2
0
2
4
Log(λ)
Coefficients
-0.2
-0.1
0.0
0.1
0.2
Log(λ)

B

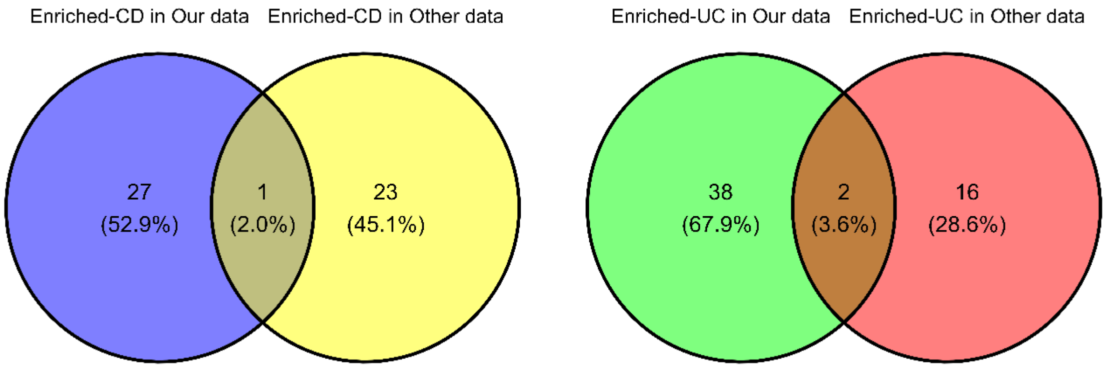

Enriched-CD in Our data
Enriched-CD in Other data
27 (52.9%)
1 (2.0%)
23 (45.1%)
Enriched-UC in Our data
Enriched-UC in Other data
38 (67.9%)
2 (3.6%)
16 (28.6%)

Fig. S7

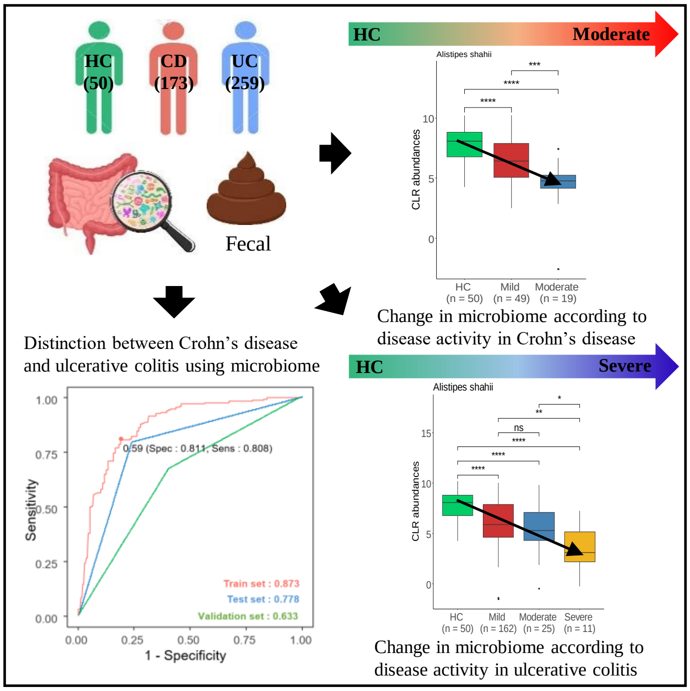
HC (50)
CD (173)
UC (259)
Fecal
Distinction between Crohn's disease and ulcerative colitis using microbiome
0.59 (Spec : 0.811, Sens : 0.808)
Train set : 0.873
Test set : 0.778
Validation set : 0.633
Sensitivity
1 - Specificity
HC
Moderate
Alistipes shahii
CLR abundances
HC (n = 50)
Mild (n = 49)
Moderate (n = 19)
Change in microbiome according to disease activity in Crohn's disease
HC
Severe
Alistipes shahii
CLR abundances
HC (n = 50)
Mild (n = 162)
Moderate (n = 25)
Severe (n = 11)
Change in microbiome according to disease activity in ulcerative colitis
