# Supplementary material for: Diagnosis of Crohn’s disease and ulcerative colitis using the microbiome
Source: BMC Microbiol. 2023 Nov 11;23:336. doi: 10.1186/s12866-023-03084-5 (PMC10640746; doi:10.1186/s12866-023-03084-5)
Supplement: Supplementary file 2 — Additional file 2: Supplementary Table 1. Association between enterotypes and IBD occurrence. [file 12866_2023_3084_MOESM2_ESM.docx]

**Supplementary information**

Supplementary Tables

Supplementary Table 1. Association between Enterotypes and the IBD occurrence.

Supplementary Table 2: Differences in the taxonomic composition of the gut microbiome in patients with CD and UC

Supplementary Table 3: Metacyc pathway of the different gut microbiomes (CD VS UC)

Supplementary Table 4: KEGG Orthology of the different gut microbiomes (CD VS UC)

Supplementary Table 5: Differences in the taxonomic composition of the gut microbiome in HC and CD

Supplementary Table 6: Differences in the taxonomic composition of the gut microbiome in mild and Moderate patients with CD

Supplementary Table 7: Correlation between microbes and severity of CD

Supplementary Table 8: Differences in the taxonomic composition of the gut microbiome in HC and UC

Supplementary Table 9: Differences in the taxonomic composition of the gut microbiome in mild and Severe patients with UC

Supplementary Table 10: Correlation between microbes and the severity of UC

Supplementary Table 11: Microbial coefficients used for modeling

Supplementary figures

Fig. S1 Enterotypes identified in individuals with HC and IBD. (A) Using principal coordinate analysis (PCoA), participants were divided into three enterotypes, with *Bacteroides, Faecalibacterium* or *Bifidobacterium* as the primary distinguishing factors. (B) Three bacteria most abundant among the three enterotypes (Wilcoxon test, *p* < 0.05)

Fig. S2 Comparison of the relative abundance of microbiota among HC, CD, and UC in phylum (A), genus (B) and species (C) based on the Kruskal-Wallis test. Each figure is shown in the order of HC, CD and UC.

Fig. S3 Comparison of the relative abundance of microbiota between disease stages (HC, mild, and moderate) in CD patients at the phylum (A), genus (B) and species (C) levels. The numbers are listed in the following order: HC, mild and moderate.

Fig. S4 Comparison of the relative abundance of microbiota between disease stages in UC patients at the levels of phylum (A), genus (B), and species (C) levels. The numbers are listed in the following order: HC, mild, moderate, and severe.

Fig. S5 Comparison of 27 supervised machine learning models for diagnosing IBD subtypes using differential abundance. (A) F1 score; (B) ROC AUC

Fig. S6 (A) Parameter adjustment of a supervised machine learning model to classify CD and UC individuals using gut microbiomes. Ten-fold cross-validation was performed to select the optimal lambda. The point where the mean square error (MSE) was minimized was designated as the best lambda (log [λ] = -2.016529). (B) Venn diagram comparing our microbiome list with that of Clonney et al.

Fig. S7 Graphical abstract

Supplementary Table 1. Association between enterotypes and IBD occurrence.

| Comparison | Group | *Bifidobacterium* | *Faecalibacterium* | *Bacteroides* | *p*-value* |
| --- | --- | --- | --- | --- | --- |
| HC vs CD | HC | 6 | 37 | 7 | < 0.001 |
|  | CD | 50 | 64 | 59 |  |
| HC vs UC | HC | 6 | 37 | 7 | < 0.001 |
|  | UC | 99 | 104 | 56 |  |
| CD vs UC | CD | 50 | 64 | 59 | 0.01 |
|  | UC | 99 | 104 | 56 |  |

*Fisher’s exact test
